# Experimental realisation of tunable ferroelectric/superconductor $({\text {B}} {\text {T}} {\text {O}}/{\text {Y}} {\text {B}}{\text {C}} {\text {O}})_{{\text {N}}}/{\text {S}}{\text {T}}{\text {O}}$ 1D photonic crystals in the whole visible spectrum

**DOI:** 10.1038/s41598-020-69811-4

**Published:** 2020-08-04

**Authors:** Luz E. González, John E. Ordoñez, Carlos A. Melo-Luna, Evelyn Mendoza, David Reyes, Gustavo Zambrano, Nelson Porras-Montenegro, Juan C. Granada, Maria E. Gómez, John H. Reina

**Affiliations:** 1 Centre for Bioinformatics and Photonics (CIBioFi), Universidad del Valle, Edificio E20 No. 1069, 760032 Cali, Colombia; 2 Solid State Theoretical Physics Group, Departamento de Física, Universidad del Valle, 760032 Cali, Colombia; 3 Facultad de Ciencias Naturales y Matemáticas, Universidad de Ibagué, 730001 Ibagué, Colombia; 4 Thin Films Group, Departamento de Física, Universidad del Valle, 760032 Cali, Colombia; 5 Quantum Technologies, Information and Complexity Group, Departamento de Física, Universidad del Valle, 760032 Cali, Colombia; 6 Centre d’Élaboration de Matériaux et d’Etudes Structurales (CEMES) CNRS-UPR 8011, 29 Rue Jeanne Marvig, 31055 Toulouse, France

**Keywords:** Condensed-matter physics, Photonic crystals, Optical spectroscopy

## Abstract

Emergent technologies that make use of novel materials and quantum properties of light states are at the forefront in the race for the physical implementation, encoding and transmission of information. Photonic crystals (PCs) enter this paradigm with optical materials that allow the control of light propagation and can be used for optical communication, and photonics and electronics integration, making use of materials ranging from semiconductors, to metals, metamaterials, and topological insulators, to mention but a few. Here, we show how designer superconductor materials integrated into PCs fabrication allow for an extraordinary reduction of electromagnetic waves damping, making possible their optimal propagation and tuning through the structure, below critical superconductor temperature. We experimentally demonstrate, for the first time, a successful integration of ferroelectric and superconductor materials into a one-dimensional (1D) PC composed of $({\text {B}}{\text {T}}{\text {O}}/{\text {Y}}{\text {B}}{\text {C}} {\text {O}})_{{\text {N}}}/{\text {S}}{\text {T}}{\text {O}}$ bilayers that work in the whole visible spectrum, and below (and above) critical superconductor temperature $T_C=80\, {\hbox {K}}$. Theoretical calculations support, for different number of bilayers *N*, the effectiveness of the produced 1D PCs and may pave the way for novel optoelectronics integration and information processing in the visible spectrum, while preserving their electric and optical properties.

## Introduction

The use of electromagnetic (EM) waves as information carriers for communication systems has been in place for many years^1,2^; as such, EM wavelengths make possible the transmission over large distances but, at the same time, they limit the amount of information they can convey by their frequency: the larger the carrier frequency, the larger the available transmission bandwidth and thus the information-carrying capacity of the communication system^3^. For this reason, quantum artificial nanostructured materials such as photonic crystals that are able to transmit at high frequencies and that concentrate the available power within the transmitted electromagnetic wave, thus giving an improved system performance, are desired^4^. PCs are artificial periodic structures characterised by a periodic variation of the refractive index with a consequent periodic spatial variation of the dielectric constant, which may be tailored to control light properties^4^. They, therefore, allow the appearance of defined frequency ranges and address the issue of forbidden/allowed propagation of electromagnetic waves. As a consequence, the control and tunability of PCs opens a new perspective for information processing and technological applications such as chips^5,6^, filters^7^, lasers^8^, waveguides^9–11^, integrated photonic circuits^12^, chemical and biological sensors^13^, and thin film photovoltaics^14,15^, to cite but a few.

Even though there exist many sort of materials used for tunable PCs^16–19^, substantial advances would be expected if superconductor properties (YBCO) could be merged with those of ferroelectric ones (BTO), on a photonic structure. As it is well known, superconductors are materials characterised for low losses and better operating characteristics than normal metals. Nowadays, they are regarded as promising quantum materials, widely used in quantum computing networks^20–23^, quantum simulators^24,25^, loss-less microwave resonators^26^, and AC Josephson junction lasers^27^. Superconductors used as building blocks for PCs fabrication have mainly two advantages over traditional materials. First, the damping (losses)-issue of electromagnetic waves presented in metals can be overcome by resorting to superconductors instead. Second, the dielectric function of a superconductor is mainly dependent on the London penetration depth, which is a function of temperature and external magnetic field^28–30^. Thus, superconductor materials represent an opportunity worthy of consideration for the implementation of tunable photonic crystals with remarkable applications as optical filters, reflectors, switches and sensing devices, and tunable resonators^31–36^, among others. On the other hand, ferroelectric thin films feature significant technological aspects such as short response time^37^, remanent polarization^38^, faster tuning compared to ferromagnetic materials^39^, smaller and lighter structures^40^, high power capacity^41^, and Pockels modulation^42^, which make of BTO a suitable material for developing high-performance electro-optical modulators^43,44^, photodetectors^45^, and novel devices such as, non-volatile optical memories^46,47^. In particular, the latter could serve in a wide range of applications, such as a high-speed optical buffer memory by means of the high broadband of optical fibers, with a significant reduction of power consumption for data processing.

Furthermore, several technical reasons back the merging of high temperature superconductor (YBCO) and ferroelectric (BTO) thin films: First, both materials have perovskite structure, good lattice matching, and chemical similarity, which facilitates a successful epitaxial growth of high-quality ferroelectric/superconductor thin films^48,49^. Second, both materials bear thermal expansion and contraction, besides severe mechanical stress, owing to variations between ambient and cryogenic temperature^48^. Third, such a nanosystem is compatible with the thin film filter technology used in the manufacturing industry of optical fibers and optoelectronic devices^50^. The relevance to technological applications of superconductor/ferroelectric photonic heterostructures has recently been pointed out in^51,52^, for the fabrication of superconductor/ferroelectric and superconductor/ferroelectric/superconductor heterostructures based on YBCO and BFO. These have produced strong and nonvolatile field effects for applications such as Josephson memory devices and ferroelectric field-effect superconducting transistors^53^, among others. Prompted by the above grounds, novel physical properties of ferroelectric and superconductor heterostructures (based on BTO and YBCO) are expected. For these reasons, and taking into consideration recent developments and interest in this type of heterostructure, in this work we highlight its applicability in photonic technologies.

Here, we provide a robust integration of ferroelectric/superconductor materials in a 1D photonic heterostructure with remarkable optical properties that work at low (below $T_C$) and above $T_C=80\, {\hbox {K}}$, which can be harnessed for optical communication and optoelectronics integration. We have realised and experimentally demonstrated the optical reliability of the fabricated nanosystem via reflectance spectrometry measurements in the whole visible range, and also theoretically modelled below and above superconductor critical temperature for small and large number of heterostructure periods, bilayers *N*. Our findings may pave a way for novel PC materials development that can be merged into photonic integrated circuits, optical filters and reflectors, or in devices for the transmission of information in the visible range at cryogenic temperatures, while preserving their electric and optical properties.

## Results and discussion

**Figure 1:**
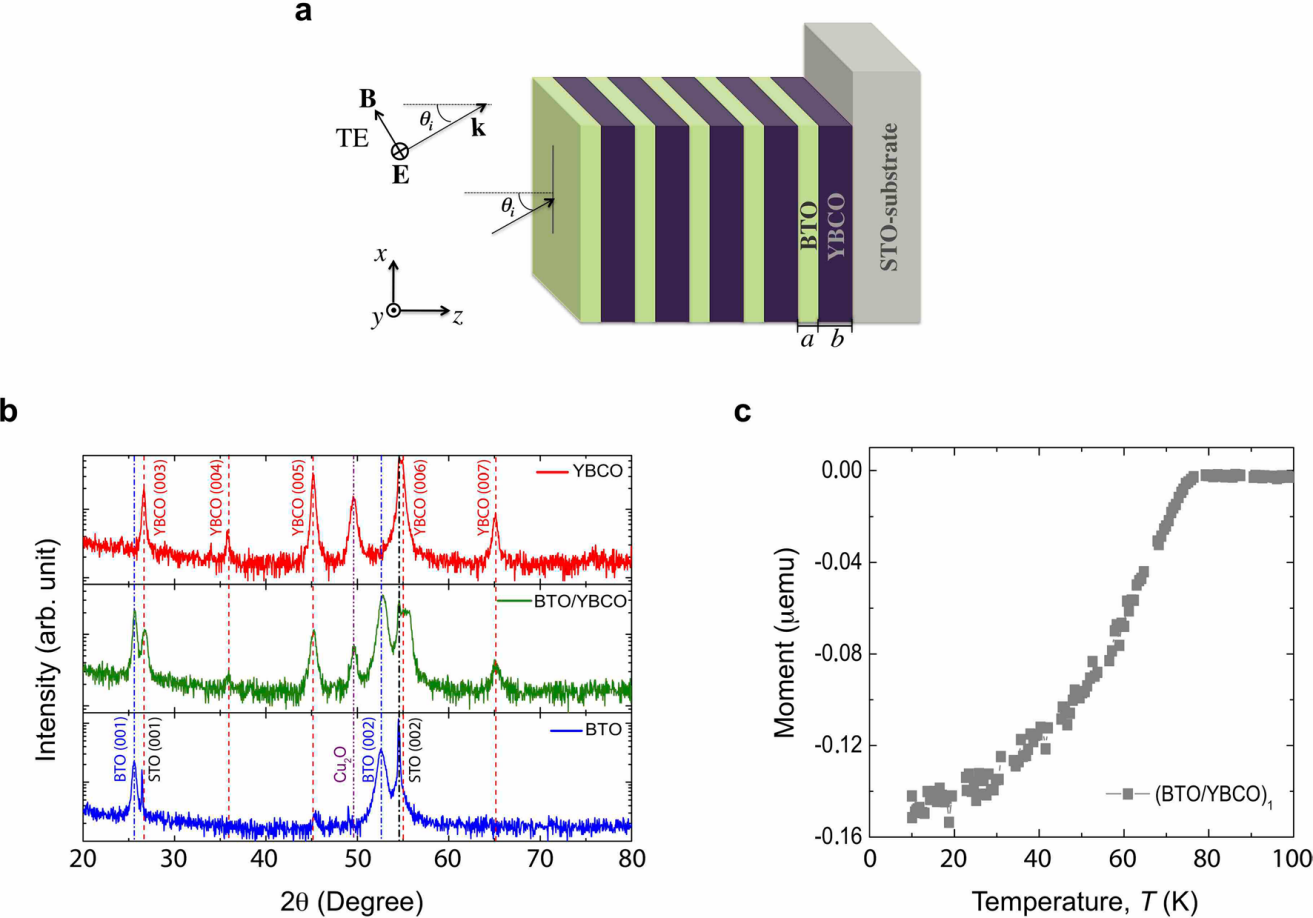
(**a**) Schematic diagram of $({\text {B}}{\text {T}}{\text {O}}/{\text {Y}}{\text {B}} {\text {C}}{\text {O}})_{N}/{\text {S}}{\text {T}}{\text {O}}$ 1D PC for $N = 1, 3, 5$ periods. The films were fabricated by RF sputtering onto a polished ${\text {Sr}}{\text {Ti}}{\text {O}}_{3}$ (001) substrate. YBCO and BTO thicknesses correspond to $b=73\, {\hbox {nm}}$ and $a=30\, {\hbox {nm}}$, respectively. (**b**) X-ray $\theta -2\theta$ scans for YBCO(70 nm)/STO film, BTO(30 nm)/YBCO(73 nm)/STO bilayer, and BTO(30 nm)/STO film. The dashed vertical lines are associated with pure phases. A small amount of ${\hbox {Cu}}_{2}{\hbox {O}}$ phase is identified for the YBCO layer. (**c**) Magnetization with temperature dependence at zero field cooling (ZFC) with an applied field $H = 1 \, {\hbox {kOe}}$ for BTO(30nm)/YBCO(73 nm)/STO for $N=1$ (gray squares).

**Figure 2:**
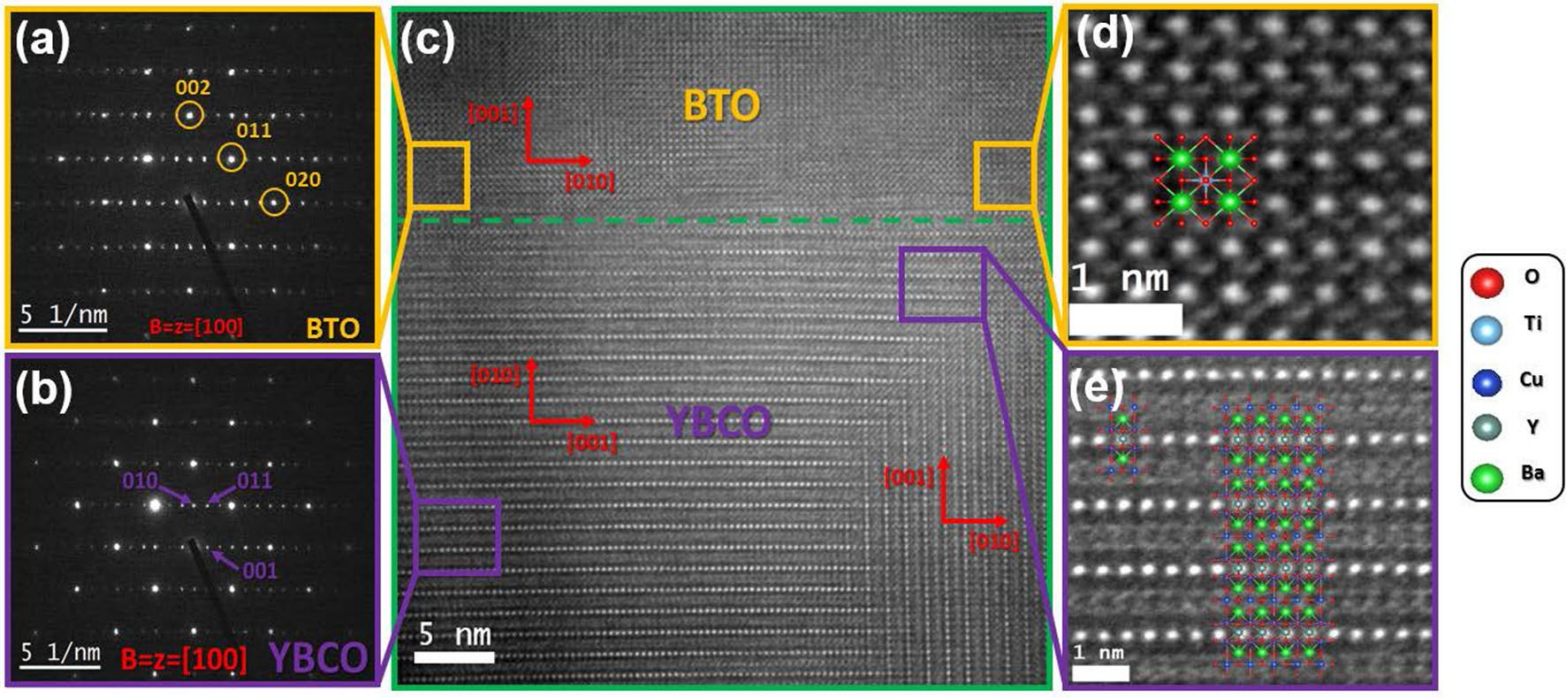
Electron diffraction pattern for (**a**) YBCO and (**b**) BTO layers with different crystal orientations are displayed. (**c**) TEM image for ${{\hbox {(BTO/YBCO)}}}_{N=1}$ with associated [001] and [010] crystal directions. Zoom of HRTEM images for (**d**) YBCO, and (**e**) BTO, respectively. A sketch of the crystal structure displays the atomic positions.

### Structural properties

Figure 1a shows a schematic diagram of three 1D photonic crystals of one-, three-, and five-pair of BTO/YBCO bilayers, fabricated by DC and RF sputtering onto polished ${\text {Sr}}{\text {Ti}}{\text {O}}_{3}$ (001) substrates. Here, *a* and *b* correspond to the thicknesses of ${\text {Ba}}{\text {Ti}}{\text {O}}_{3}$ and ${\text {Y}}{\text {Ba}}_{2}{\text {Cu}}_{3}{\text {O}}_{7}$, $\theta _{i}$ denotes the angle with the *z*-axis defined in the range of $0^{\circ } \sim \pm 90^{\circ }$, *xz* is the plane of incidence, and the direction of $\mathbf{E} \times \mathbf{B}$ is given by the incident wave vector **k**, where **E** and **B** represent the electric and magnetic fields, respectively. Figure 1b displays the out-of-plane XRD $\theta$-$2\theta$ scans for the YBCO(70 nm)/STO film, the BTO(30 nm)/YBCO(73 nm)/STO bilayer, and the BTO(30 nm)/STO film. YBCO and BTO peaks associated with the (001) direction were identified for reflections from $20^{\circ }$ to $80^{\circ }$. The observed peaks in the individual layers are indexed in the bilayer, and indicate a textured growth of both samples. A minor ${\text {Cu}}_{2}{\text {O}}$ phase was identified in our sample, this impurity is typical for YBCO^54,55^. The following lattice parameters were obtained: $\textit{a}_{BTO}=4.035 \, {\AA }$, $\textit{a}_{YBCO}= 3.878 \, {\AA }$ in films, while $\textit{a}_{BTO}=4.040 \, {\AA }$ and $\textit{a}_{YBCO}= 3.867 \, {\AA }$ for the BTO/YBCO bilayer. For a bilayer, no considerable displacement of peaks was observed compared to the YBCO and BTO films grown under identical parameters, as shown by the dashed vertical lines in the X-ray $\theta -2\theta$ scans of Fig. 1b. Hence, there is no effect due to the BTO layer on the position of the Bragg reflection peaks for the YBCO layer.

With the aim to corroborate the SC/ferroelectric PC formation, we first performed a resistivity measurement as a function of temperature for an YBCO film to confirm the superconductor state. As expected, a traditional resistance with temperature dependence was obtained, with a superconducting temperature $T_{SC}\sim 85 \, {\hbox {K}}$ for the BTO/YBCO film. During the ${{\hbox {(BTO/YBCO)}}}_{N}$-PC structure measurements, the YBCO layer was, at some point, exposed to laser irradiation at different wavelengths. At a first glance, this could imply the possibility of occurrence of a breakdown of the Cooper pairs during the radiation-matter interaction (e.g., in YBCO intergranular films, the radiation damage requires $\sim$ 1 eV/atom)^56–59^. We performed a resistance measurement for the YBCO/STO film whilst the sample was exposed to a coherent laser radiation source, in the 500–800 nm range. For YBCO, the superconducting gap equals 30 meV and the laser radiation energy which would break down the Cooper pairs is far from this, in the range between 2.5 eV (800 nm) and 3.0 eV (400 nm)^60^. Furthermore, the sample is irradiated with a power below 10 mW, hence the sample remains in the superconducting state, even though the energy of the laser radiation is a hundred times greater than the superconducting gap. This said, a slight displacement associated with thermal effects was found when the laser is on ($T_{SC}\sim 87 \, {\hbox {K}}$), i.e., in our case, the radiation-matter interaction does not significantly influence the YBCO superconductivity and, indeed, allows for a PC in the superconductor state. For $({\text {B}}{\text {T}}{\text {O}}/{\text {Y}}{\text {B}}{\text {C}}{\text {O}})_{N}$ multilayers, the superconductivity state is indirectly measured from YBCO diamagnetism^61^. In Fig. 1c, the temperature-dependent magnetisation for the $({\text {B}}{\text {T}}{\text {O}}/{\text {Y}}{\text {B}}{\text {C}}{\text {O}})_{1}$ bilayer is depicted, and a superconducting transition temperature decrease to $T_{SC}\sim 70 \, {\hbox {K}}$ is reached. This decrease can be associated with different mechanisms such as tensile/compressive strain in BTO/YBCO interfaces, interface atom migration during the film growth^62^, oxygen losses^63,64^, dislocations^65^, and Cooper pair breaks^66–68^, among others^69^. However, we emphasize that the PC superconducting state is preserved.

The analysis of the BTO/YBCO interface by means of transmission electron microscopy (TEM) shows the following. The electron diffraction pattern for the YBCO and BTO layers is presented in Fig. 2a, b (the electron diffraction patterns were rotated $90^{\circ }$ to match with the high-resolution image orientations). The lamella was slightly tilted to achieve the closest zone axis for the STO crystal. Figure 2c, a high-resolution image, shows the YBCO/BTO bilayer with a flat interface and layer orientation, and the YBCO layer mainly grows in the [010] direction; a few grains growing along the [001] orientation were found. Additionally, the BTO growth along the [001] orientation agrees with the STO substrate growth direction. Finally, Fig. 2d and e display a high-resolution image for YBCO and BTO, respectively, where the atomic columns are visible for some of the atoms. The arrangement of the different atoms is represented by the unit cell for BTO (Fig. 2d) and YBCO (Fig. 2e) layers. It can clearly be seen, for example, that for YBCO a supercell composed of 16 unit cells shows a very good matching between the crystal structure and the high-resolution image.

**Figure 3:**
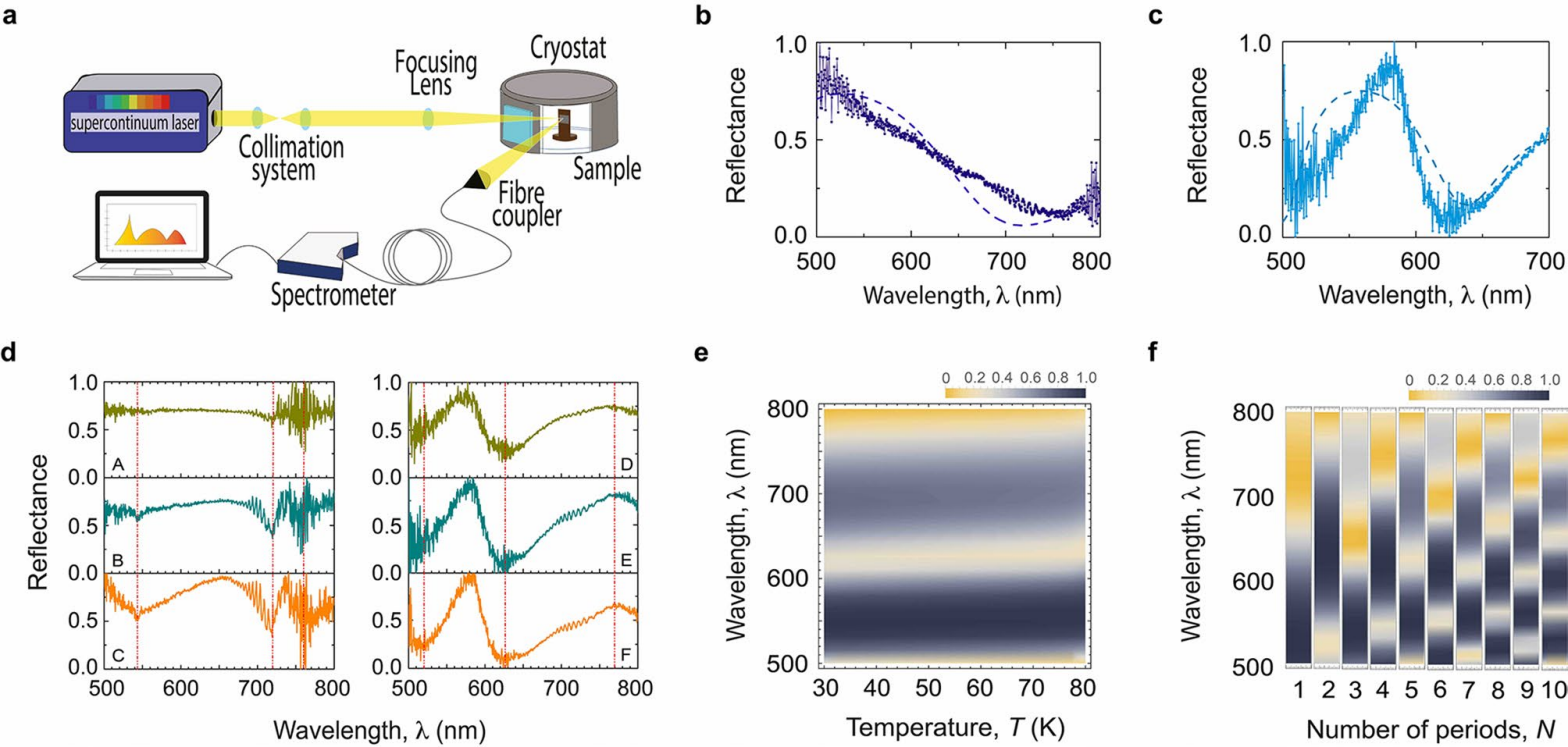
(**a**) Optical setup. A supercontinuum laser beam in the wavelength range between 400 and 800 nm, collimated with a Galilean telescope lenses setup, is sent to the multilayer $({\text {B}}{\text {T}}{\text {O}}/{\text {Y}}{\text {B}}{\text {C}}{\text {O}})_{{\text {N}}}$ sample inside a cryostat, at a controllable temperature, toward mirrors and a focusing lens. The reflection from the sample is collected by a fiber coupler into a multimode fiber towards the spectrometer and the response is analysed in a workstation. (**b**), (**c**) Reflectance response of $({\text {B}}{\text {T}}{\text {O}}/{\text {Y}}{\text {B}}{\text {C}} {\text {O}})_{{\text {N}}}/{\text {S}}{\text {T}}{\text {O}}$ 1D PC for $N=1$, and 5, respectively. Continuous and dashed curves correspond to experimental and theoretical results at $T=50\, {\hbox {K}}$, respectively. (**d**) Reflectance response of $({\text {B}}{\text {T}}{\text {O}}/{\text {Y}}{\text {B}}{\text {C}} {\text {O}})_{{\text {N}}}/{\text {S}}{\text {T}}{\text {O}}$ 1D PC for (A) $T=80\, {\hbox {K}}$, (B) $T=50\, {\hbox {K}}$, and (C) $T=30\, {\hbox {K}}$, for $N = 3$, and panels (D) $T=80\, {\hbox {K}}$, (E) $T=50\, {\hbox {K}}$, and (F) $T=30\, {\hbox {K}}$, for $N = 5$. (**e**) Simulated photonic band structure of the $({\text {B}}{\text {T}}{\text {O}}/{\text {Y}}{\text {B}}{\text {C}} {\text {O}})_{5}/{\text {S}}{\text {T}}{\text {O}}$ 1D PC as a function of wavelength and temperature *T*, from $T=30\, {\hbox {K}}$ to 80 K. The dark areas correspond to the high-reflectance ranges and yellow regions indicate high transmission bands where radiation passes through the structure. (**f**) Simulated photonic band structure of the $({\text {B}}{\text {T}}{\text {O}}/{\text {Y}}{\text {B}} {\text {C}}{\text {O}})_{N}/{\text {S}}{\text {T}}{\text {O}}$ 1D PC as a function of wavelength and number of periods *N*. The width of each bar corresponds to a temperature range between 50 and 60 K.

### Optical response: theory and experiment

Figure 3a shows the schematics of the experimental setup used to measure the optical response of the $({\text {B}}{\text {T}}{\text {O}}/{\text {Y}}{\text {B}} {\text {C}}{\text {O}})_{N}/{\text {S}}{\text {T}}{\text {O}}$ 1D PC (described in the Methods section). Figure 3b, c display the effect of the number of periods on the reflectance spectra. Continuous and dashed curves correspond to the experimental and theoretical results obtained when *N* is equal to 1 and 5, and the temperature is kept constant at $T=50\, {\hbox {K}}$, respectively. Even though measured spectra are in agreement with the theoretical predictions, the overall behaviour presents a considerable number of resonant peaks and a small variation in reflectance intensity. Based on the TEM analysis of Fig. 2, we associate these peaks with the possible non-uniformity of the thickness and the additional presence of a few YBCO grains grown along the [001] direction. Additionally, correspondence between the number of interference fringes (or bands) and the number of periods of bilayers in the structure is evidenced. These fringes are likely the result of the interference of incident light beams partially reflected and transmitted at the interfaces between layers.

To gain further insight into the optical response of our structure, we used the transfer matrix method to calculate the reflectance spectra^70,71^, and the two-fluid model^72^, to consider the contribution of the superconductor (YBCO) to the dielectric response of the PC (see the Methods section). As described ahead, this model allowed us to theoretically explain the reflectance found experimentally. In a previous work^71^, we theoretically studied the transmission of the superconductor PC as a function of the wavelength for different temperatures. We found no noticeable changes in the transmission spectra with temperature, but did find the existence of small shifts in the bands. With the aim to compare with experimental results, in Fig. 3d, we plot, in six panels, the temperature effect on the reflectance spectra measured. From panels (A), (B), and (C), where the temperature varied from 80 K, 50 K to 30 K for $N = 3$, an almost negligible shift is perceived in the wavelength ranges where the transmission bands are present. Similar features were obtained in panels (D), (E) and (F), for $N = 5$ with $T=80~{\hbox {K}}$, 50 K and 30  K, respectively. Figure  3d indicates with vertical red lines specific wavelengths ($\lambda = 543$, 720, and 760 nm for $N=3$, and $\lambda = 520$, 625, and 770 nm for $N=5$) to get a better visualisation of these findings. In addition, in Fig. 3e, we show the $({\text {B}}{\text {T}}{\text {O}}/{\text {Y}}{\text {B}}{\text {C}}{\text {O}})_{5}/ {\text {S}}{\text {T}}{\text {O}}$ 1D PC optical response as a function of wavelength and temperature *T*, from $T=30~{\hbox {K}}$ to 80 K, below the critical temperature of the superconductor. The dark areas correspond to the high-reflectance ranges, while yellow areas, indicate high transmission ranges where radiation passes through the structure. We obtain a negligible shift as the temperature increases. We associate our findings with the slight decrease suffered by the superconductor dielectric constant as the wavelength increases. If this change were appreciable, the reflectance should suffer a displacement, in agreement with the electromagnetic variational principle^4^. Thus, our results demonstrate the effectiveness of the $({\text {B}}{\text {T}}{\text {O}}/{\text {Y}}{\text {B}}{\text {C}} {\text {O}})_{N}/{\text {S}}{\text {T}}{\text {O}}$ 1D PCs implemented as a promising choice in the design of optical transmitters/reflectors below and above critical superconductor temperature. The fact that temperature does not significantly affect the operation frequencies of the bands becomes an advantage, given that high reflectances can be achieved in that whole range of temperatures.

In order to examine in detail the band dependence on the number of periods, in Fig. 3e we plot the simulated optical response in the whole range from $N = 1$ to 10. As in the previous case, dark areas correspond to the high-reflectance ranges, while yellow areas indicate transmission ranges where radiation passes through the structure. It is noticeable for $N = 1$ that reflectance decreases from 500 nm to approximately 610 nm (see Fig. 3b), which is observed in the optical response of Fig. 3f as the change from dark to yellow region. In the case $N = 5$, two low-reflectance ranges are seen around 510 nm and 620 nm (Fig. 3c), which is completely in agreement with the theory. We have extended our results up to $N = 10$, to show the sensitivity of the transmission/reflection bands with *N*: the larger *N*, the lower the wavelengths of the photonic bands, and their optical response experiences a switch for the EM waves propagation from forbidden to allowed frequency ranges at a given wavelength. Interestingly, such structured bands become narrower as *N* changes from an even to an odd number, a mechanism that could be exploited as an optical switch.

**Figure 4:**
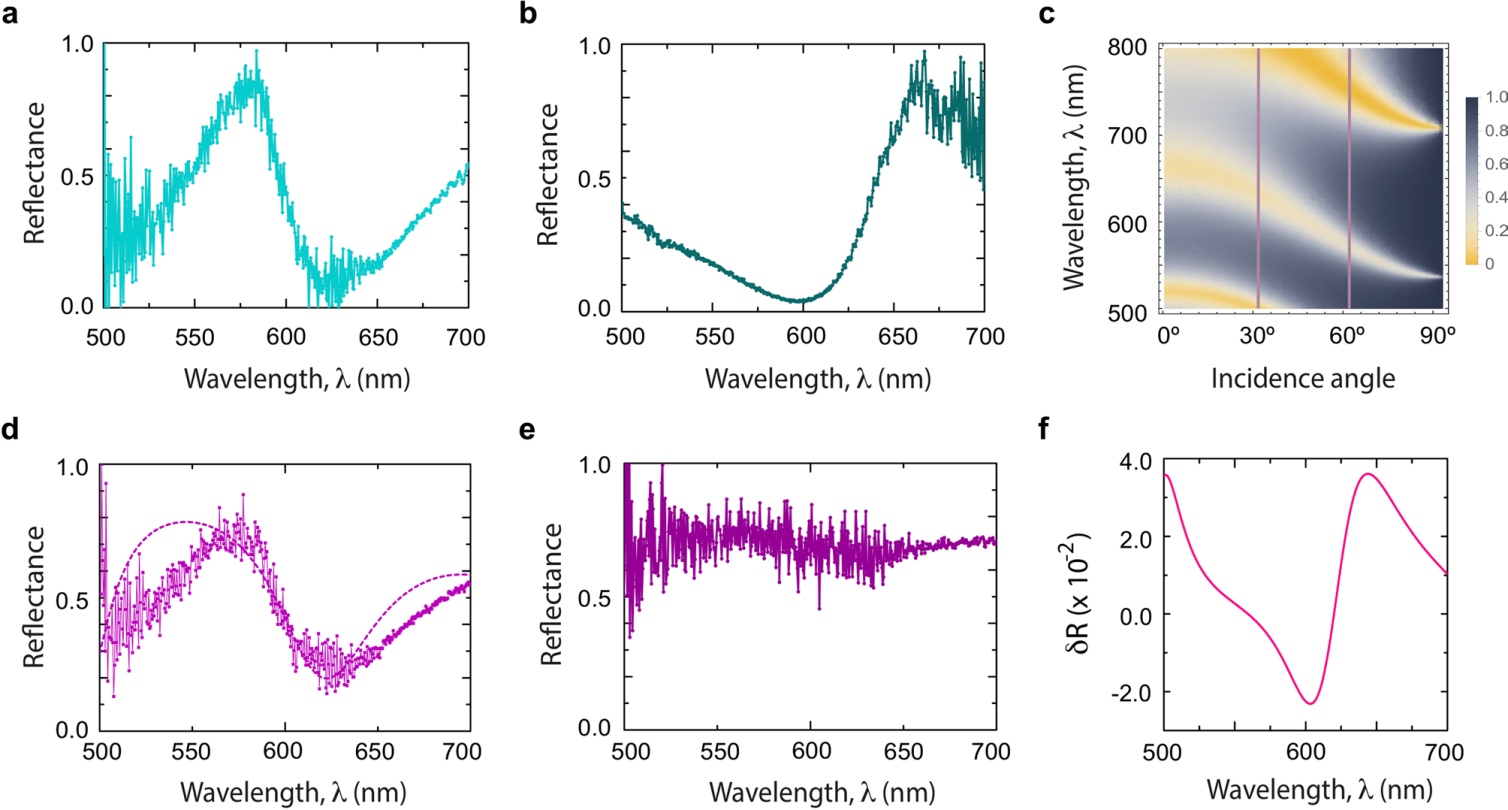
Panels (**a**) and (**b**) display the measured reflectance of $({\text {B}}{\text {T}}{\text {O}}/{\text {Y}}{\text {B}}{\text {C}} {\text {O}})_{5}/{\text {S}}{\text {T}}{\text {O}}$ 1D PC at $T= 50\, {\hbox {K}}$ and incident angles of $35^{\circ }$ and $65^{\circ }$ for TE polarization, respectively. (**c**) Optical response for TE polarization in the whole range of incident angles, also at $T= 50\, {\hbox {K}}$. The dark areas correspond to the high-reflectance ranges and yellow regions indicate high transmission bands where radiation passes through the structure. Magenta vertical lines in the figure are a guide to the eye, and correspond to the results for $35^{\circ }$ (Fig. 4a) and $65^{\circ }$ (Fig. 4b), respectively. (**d**) Experimental (continuous curve) and theoretical (dashed curve) reflectance spectra for $({\text {B}}{\text {T}}{\text {O}}/{\text {Y}}{\text {B}}{\text {C}} {\text {O}})_{5}/{\text {S}}{\text {T}}{\text {O}}$ 1D PC at $T=80\, {\hbox {K}}$, show the agreement between experiment and the theory based on the Drude model (Eq. 10). (**e**) $({\text {B}}{\text {T}}{\text {O}}/{\text {Y}}{\text {B}}{\text {C}} {\text {O}})_{5}/{\text {S}}{\text {T}}{\text {O}}$ experimental spectrum at $T=100\, {\hbox {K}}$, above superconductor critical temperature $T_{c}$. (**f**) Contribution due to superconductor carriers on the optical response of the $({\text {B}}{\text {T}}{\text {O}}/{\text {Y}}{\text {B}} {\text {C}}{\text {O}})_{5}/{\text {S}}{\text {T}}{\text {O}}$ 1D PC.

Figure 4a, b plot the measured reflectance for $({\text {B}}{\text {T}}{\text {O}}/{\text {Y}}{\text {B}}{\text {C}} {\text {O}})_{5}/{\text {S}}{\text {T}}{\text {O}}$ 1D PC in the wavelength range from 500 to 700 nm at $T=50\, {\hbox {K}}$ and incident angles of $35^{\circ }$ and $65^{\circ }$ for TE polarization. Two high reflectance regions were found around 570 nm and 650 nm at $35^{\circ }$ and $65^{\circ }$, respectively. For better understanding of the reflectance behaviour, we have displayed in Fig. 4c, the spectra as a function of the whole range of incident angles for TE polarization. One important feature found in our results related to the reflection from a finite multilayer is its sensitive response to the light incidence angle^4^, observed in the continuous displacement of the bands to shorter wavelength as the incident angle increases. On the other hand, as the incidence angle approaches $90^{\circ }$, the reflection coefficient tends to 1, a result in agreement with the fact that a wave that impinges with a right angle moves parallel to the separation surface of the two media, and therefore, its energy is not transmitted through the surface. The results for $35^{\circ }$ and $65^{\circ }$ (magenta vertical lines) are indicated in the figure, whose bands match perfectly well with measured spectra (Fig. 4a, b). Figure 4c also evidences the existence of a third transmission band for wavelengths above 700 nm. These results allow us to conclude that there exist wavelength ranges where the radiation does not pass through the PC under any incident angle, results that can be applied, for example, for the tuning of optical transmitters/reflectors fabricated to operate below critical superconductor temperature, whose response is sensitive to the light incidence angle.

For the sake of completeness, we calculate the optical response of $({\text {B}}{\text {T}}{\text {O}}/{\text {Y}}{\text {B}}{\text {C}} {\text {O}})_{5}/{\text {S}}{\text {T}}{\text {O}}$ 1D PC when the ${\text {Y}}{\text {B}}{\text {C}}{\text {O}}$ is in the non-superconducting state, above $T_{c}$. In this case, the dielectric function is described by the Drude model of a metal given by Eq. (10). As is well known, for temperatures below $T_{c}$ there is no resistance, and for temperatures above $T_{c}$ there is a non-zero resistance that varies linearly with the temperature. Resistivity measurements were performed with (and without) applied laser field irradiation on the $({\text {B}}{\text {T}}{\text {O}}/{\text {Y}} {\text {B}}{\text {C}}{\text {O}})_{N}$ 1D PCs. The main goal of this measurement was to confirm that the superconductor (${\text {Y}}{\text {Ba}}_{2}{\text {Cu}}_{3}{\text {O}}_{7}$) remains in its superconducting state under applied radiation. Even though the superconductor was radiated at frequencies above its superconductor gap ($\sim$ 8 THz), the 10 mW laser power in the spectral band of 450–750 nm is not enough to destroy the superconducting state.

**Figure 5:**
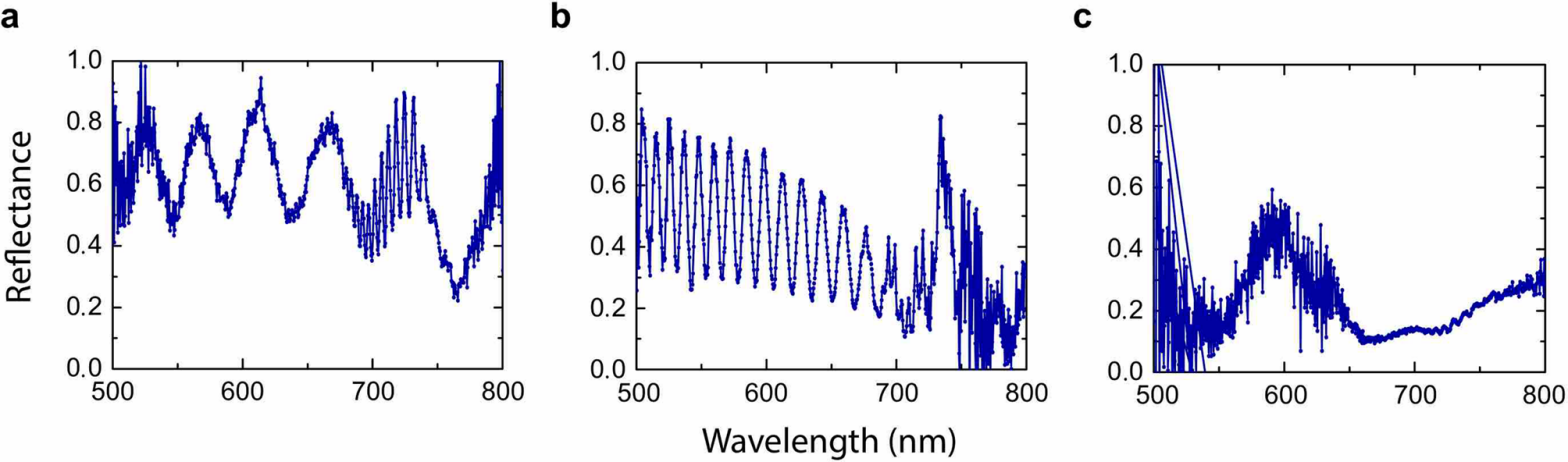
Reflectance spectra for $({\text {B}}{\text {T}}{\text {O}}/{\text {Y}}{\text {B}} {\text {C}}{\text {O}})_{{\text {N}}}/{\text {S}}{\text {T}}{\text {O}}$ 1D PCs for periods $N= 1$ (**a**), 3 (**b**), and 5 (**c**), at $T=20\, {\hbox {K}}$, in the 500–800 nm spectral band.

Accordingly, in Eq. (11), the damping $\gamma$ varies linearly with temperature in accordance with $\rho _{0}=(3.74\times 10^{-9})T+(6.90\times 10^{-7})$ for $T>T_{c}$, which implies that Eq. (10) is temperature-dependent. The calculated and measured reflectance are shown in Fig. 4d, and the behaviour predicted by the Drude model is in excellent agreement with our experimental results on the reflection of electromagnetic waves at the interface between the ferroelectric and the superconductor in normal state. When the electromagnetic wave impinges on the conductor surface it induces a conduction current, which leads to a swift damping of the field inside the conductor. This is why metals have excellent reflecting properties (i.e., reflectance close to unity) and are broadly used in mirrors or optical reflectors. According to the above, a high reflectance would be expected in the PC now composed of the superconductor in the normal state. However, as it is observed in the Fig. 4d, e, the intensity of the reflectance increases with respect to the previous cases, but it is not total because about 60% and 40% of the light is still transmitted through the structure at certain wavelengths between 500 nm and 700 nm, at $T=80\, {\hbox {K}}$, and about 80% at $T=100\, {\hbox {K}}$, respectively. It is important to point out that reflectivity also depends on the thickness of the layers, and our findings clearly illustrate such thickness dependence^73,74^.

In order to analyse the superelectrons contribution on the optical response of the $({\text {B}}{\text {T}}{\text {O}}/{\text {Y}}{\text {B}}{\text {C}}{\text {O}})_{5}/{\text {S}}{\text {T}}{\text {O}}$ 1D PC, in Fig. 4f, we report the difference between the reflectance coefficients ($\delta$R) calculated from the subtraction of the reflectance spectra with and without the superelectron effect according to Eqs. (9) and (10). This result allows us to approximate the magnitude order of the contribution made by the Cooper pairs in the mixed state of the superconductor, below critical temperature, to be in the order of $\sim 10^{-2}$, and corroborate their significant contribution to the optical response of the superconductor. From the reflectance spectra at $T=100\, {\hbox {K}}$ ($T>T_{c}$), and at $T=80\, {\hbox {K}}$, 50 K and 30 K ($T\le T_{c}$), as can be seen in Fig.  3d, panels D, E, and F, for $N = 5$, we get a decreasing of the reflectance at low temperatures for wavelengths between 500–550 nm, and 600–650 nm, which implies that the main contribution to the light transmission in the structure is due to the superelectrons. This result highlights the important role played by the superconductor in the optical response of the PC. On the other hand, it is worth noting that the Drude model matches perfectly well with the experimental spectrum at 80 K, that is, the temperature around which the state transition occurs; however, and as expected, this model does not adjust the measurements above $T_{c}$ (e.g., at $T=100\, {\hbox {K}}$). Although the Drude model is presented for temperatures above critical temperature, it is evident that for $T>T_{c}$, additional contributions exist to the electronic properties, affecting the optical response.

Finally, reflectance spectra of $({\text {B}}{\text {T}}{\text {O}}/{\text {Y}}{\text {B}}{\text {C}}{\text {O}})_{N}/{\text {S}}{\text {T}}{\text {O}}$ heterostructures for $N = 1, 3, 5$, at $T=20\, {\hbox {K}}$ are shown in Fig. 5. The reflection spectra for $N=1$ and $N=3$ structures at $T=20\, {\hbox {K}}$ clearly show a constructive interference effect between the incoming light in the YBCO and the reflected light on the YBCO/BTO interface, bearing in mind that the BTO refraction index is larger than that of the YBCO. This is clearly evidenced since the reflected spectra of the $N=3$ structure shows that a maximum (minimum) takes place at each third of the wavelength of the $N=1$ heterostructure. On the other hand, the reflectance spectrum for $N=5$ begins to exhibit the whole reflectance behaviour of the 1D photonic crystal, as reported in^71^.

## Conclusions

We have succeeded at experimentally realising ferroelectric/superconductor 1D photonic crystals as suitable engineered nanosystems for tuning and controlling electromagnetic wave propagation in a wide region of the visible spectrum. We were able to fabricate 1D photonic crystals of $N=1, 3$ and 5 pairs of BTO/YBCO bilayers by DC and RF sputtering, onto polished ${\text {SrTiO}}_{3}$ (001) substrates, and studied the effects due to temperature and direction of the incident radiation. We have experimentally demonstrated how to tailor the number of photonic bands as a function of *N*, and have also been able to quantify and predict, for any *N*, the frequency range sensitivity and optical properties of the PCs with the direction of the incident EM waves—the larger the angle of incidence the shorter the wavelength and the bigger the width of the transmission/reflection bands. A key result from an operational point of view is that temperature does not significantly affect the frequency range of the transmission bands, which can be advantageous because this can enable either a high or low reflectance, in the whole range of studied temperatures (20–100 K). The contribution made by the Cooper pairs in the mixed state of the superconductor to the PC optical response is of the order of $10^{-2}$: the superelectrons are the most relevant contribution to the light transmission in the structure, at tested wavelengths between 500–550 nm, and 600–650 nm. Finally, and based on the PCs here implemented, several strategies for the development of quantum materials and possible novel optical filters and reflectors, below critical superconductor temperature and different properties of incident light, have been proposed.

## Methods

### Photonic crystal fabrication

The $({\text {B}}{\text {T}}{\text {O}}(30~{\text {nm}})/{\text {Y}} {\text {B}}{\text {C}}{\text {O}}(73~{\text {nm}}))_{N=1,3,5}$ multilayers were grown on a (001) STO substrate by DC/RF sputtering technique in a pure oxygen atmosphere and high pressure ($\sim$ 3.0 mBar), with a substrate temperature of $830^{\circ } {\hbox {C}}$. Power density of 7 ${{\hbox {W/cm}}}^{2}$ and 12 ${{\hbox {W/cm}}}^{2}$ were used for YBCO and BTO targets, respectively. X-ray diffraction was used to perform structural characterisation using a ${\hbox {Co-K}}{\alpha }=1.79 \, {\AA }$ wavelength. In addition to the local interface analysis, transmission electron microscopy (TEM) was employed. Cross-sectional lamella was prepared from the thin films deposited using a dual beam FEI Helios Nanolab 600i. Initially, the film surface was coated with a platinum layer on a sputtering to avoid charge accumulation during the process. Then, two platinum layers were applied to protect and avoid damages on the thin films. The lamella was extracted and submitted to a thinning process which ended with the use of a low energy beam to minimize amorphization. Finally, the lamella is placed onto a TEM grid. A Hitachi TEM-microscope HF3300C was operated at 300 kV to acquire the high resolution images of the cross-sectional lamella. The sample was tilted until the nearest zone axis of STO was parallel to the electron beam. Then, high resolution images were acquired at the different layers and interfaces. In addition, electron diffraction patterns were obtained to identify the crystallographic orientation of the layers with respect to the substrate. The electrical properties and the superconductor transition temperature ($T_{C}$) were studied by using the traditional four-point technique. Resistance as a function of temperature was measured in the 20–100 K range by using silver paint and copper wires. Considering that the top layer was always the BTO layer in the $({\text {B}}{\text {T}}{\text {O}}/{\text {Y}}{\text {B}}{\text {C}} {\text {O}})_{N}/{\text {S}}{\text {T}}{\text {O}}$ multilayer array, we also analysed its diamagnetic properties in order to identify $T_{c}$ through thermal demagnetization measurements, by using a PPMS Quantum Design from 10 to 300 K.

### Wide range laser reflectance measurements

A supercontinuum fiber laser (Fyla STC 1000) and a monochromator (Fyla TW) were employed to irradiate the sample in the 400-800 nm spectral range. The beam path crosses through a telescope arrangement with a magnification factor of 1$\times$ to collimate the laser and control the beam divergence. The light is addressed with aluminum mirrors (95% of reflectance) perpendicularly towards the cryostat (Cryostat Advanced Research System) where the sample is placed under a vacuum of $10^{-5}$ bar and a controlled temperature down to 10 K. The reflection angle is controlled with a homemade sample copper holder inside the cryostat, and set to $35^{\circ }$ and $65^{\circ }$ for taking different set of measurements. The reflecting path is collected with a collimation lens and coupled to a multimode optical quartz fiber $400 \, \upmu {\hbox {m}}$ in diameter. The fiber is connected to a spectrometer (HD 4000, Ocean Optics) that finally displays the spectra in a workstation. The reference spectra for the reflectance calculations were taken at high temperatures for which the reflection of the sample is higher than all the others.

### 1D photonic crystal transfer matrix calculation

The studied 1D photonic superlattice has a period *d*, and is composed of alternating layers of a dielectric material ${\text {Ba}}{\text {Ti}}{\text {O}}_{3}$ and a superconductor ${\text {Y}}{\text {Ba}}_{2}{\text {Cu}}_{3}{\text {O}}_{7}$, whose widths are labeled as *a* and *b*, respectively. The propagation of an in-plane linearly polarized electromagnetic field is of the form $\mathbf {E}\left( z,t\right) =E\left( z\right) e^{-i\omega {t}}{\hat{x}}$, along the z-axis (see Fig. 1a). By using Maxwell’s equation for linear and isotropic media, it is demonstrated that the amplitude of the electric field *E*(*z*) satisfies^71,75^

1 $$\begin{aligned} \frac{d}{d{z}}\left[ \frac{1}{\textit{n}\left( z\right) \textit{Z} \left( z\right) }\frac{\textit{dE}\left( z\right) }{\textit{dz}}\right] = -\frac{n\left( z\right) }{Z\left( z\right) }\frac{\omega ^{2}}{c^{2}}E\left( z\right) , \end{aligned}$$

where *c* is the vacuum speed of light, $\textit{n}\left( z\right) = \sqrt{\epsilon \left( z\right) }\sqrt{\mu \left( z\right) }$ and $\textit{Z}\left( z\right) =\sqrt{\mu \left( z\right) }/\sqrt{\epsilon \left( z\right) }$ are respectively, the refraction index and the impedance of each layer material. For a photonic crystal composed of alternating layers of two different materials, Eq. (1) must be solved by assuming both, the electric field and its first derivative continuous across an interface, which means that the two-component function $$\psi \left( z\right) = \begin{pmatrix} \textit{E}_{z}\\ \frac{1}{nZ}\frac{dE}{dz} \end{pmatrix}$$ is continuous through the photonic structure. This condition may be conveniently written by means of a transfer matrix as $\psi \left( z\right) = M_{i}\left( z-z_{0}\right) \psi \left( z_{0}\right)$, where

2 $$\begin{aligned} M_{i}\left( z\right) = \begin{pmatrix} \cos \left( \frac{\omega \left| n_{i}\right| }{c}z\right) &{} \frac{n_{i}}{\left| n_{i}\right| }\frac{cZ_{i}}{\omega }\sin \left( \frac{\omega \left| n_{i}\right| }{c}z\right) \\ \\ -\frac{\left| n_{i}\right| }{n_{i}}\frac{\omega }{cZ_{i}} \sin \left( \frac{\omega \left| n_{i}\right| }{c}z\right) &{} \cos \left( \frac{\omega \left| n_{i}\right| }{c}z\right) \end{pmatrix} \end{aligned}$$

in such a way that $\psi \left( \pm \frac{a+b}{2}\right)$ may be written as

3 $$\begin{aligned} \psi \left( \pm \frac{a+b}{2}\right) = M_{T}\left( \pm {a},\pm {b}\right) \psi \left( 0\right) , \end{aligned}$$

with

4 $$\begin{aligned} M_{T}\left( \pm {a},\pm {b}\right) =M_{2}\left( \pm \frac{b}{2}\right) M_{1} \left( \pm \frac{a}{2}\right) = \begin{pmatrix} P&{} \pm {Q}\\ \pm {R}&{}S \end{pmatrix}. \end{aligned}$$

The reflection coefficients are calculated by

5 $$\begin{aligned} R_{N} = \left| \frac{M_{21}}{M_{22}}\right| ^{2}, \end{aligned}$$

considering the corresponding transfer matrix for *N* periods, which can be written as^70^

6 $$\begin{aligned} M_{N}= \begin{pmatrix} P U_{N-1}- U_{N-2}&{} Q U_{N-1}\\ \\ R U_{N-1} &{}S U_{N-1}- U_{N-2} \end{pmatrix}, \end{aligned}$$

where *P*, *Q*, *R* and *S* are the elements of the transfer matrix for $N=1$. Here, $U_{N}=U_{N}(q)=\frac{\sin \left( (N+1)qd\right) }{\sin {qd}}$ are the second-order Chebyshev polynomials.

### The two-fluid model

The two-fluid model is often used to describe the behaviour of a superconductor at nonzero temperature. It consists of two distinct noninteracting fluids of electrons that carry current, where each fluid follows two parallel channels, one superconductor and one normal. Accordingly, when a material is superconducting, some of the electrons will be superconductors and some will still be normal electrons. Thus, there will be a mixture of superelectrons and normal electrons. For these reason, we can model the total conductivity as follows: for $T \le T_{c}$, as the sum of the normal conductivity maintained by unpaired electrons, and the superconducting conductivity maintained by superelectrons; and for $T > T_{c}$, by the Drude conductivity for a normal metal. More explicitly, it can be written as^72^:7
where $\omega$ is the EM wave frequency, $\mu _{0}$ is the permeability of free space; *n*, *q*, and *m* are respectively the density, the charge, and the mass of the carrriers, $\tau$ denotes the scattering time of electrons. The damping frequency $\gamma =\frac{1}{\tau }$, $f_{n}={ \left( \frac{ T }{ { T }_{ c } } \right) }^{ p }$ gives the density of normal state electrons over the total number of electrons, and $\lambda _{L}$ is the temperature-dependent penetration depth^72^:

8 $$\begin{aligned} {\lambda }_{ L }= \frac{ { \lambda }_{ 0 } }{ \sqrt{ 1-{ \left( \frac{ T }{ { T }_{ c } } \right) }^{ p } } }, \end{aligned}$$

where $\lambda _{ 0 }$ is the value of the penetration depth at zero temperature, and the exponent *p* corresponds to 2 and 4 for high and low temperature superconductors, respectively.

The dielectric response of a given material is introduced by means of the electric permittivity. This parameter is in general a complex number, $\epsilon =\epsilon _{r}+i\epsilon _{i}$, where the imaginary part $\epsilon _{i}$ accounts for electromagnetic losses in the material and is closely related to the current inside the material through the complex conductivity $\sigma (\omega )$, such that $\epsilon = \epsilon (\omega ) = \epsilon _{\infty }+i \frac{\sigma (\omega )}{\epsilon _{0}\omega }$, where $\epsilon _{\infty }$ is the dielectric function at high frequencies and $\epsilon _{0}$ the permittivity of free space^76^. By replacing Eq. (7) into $\epsilon (\omega )$, we arrive at the dielectric function of the superconductor

9 $$\begin{aligned} \epsilon (\omega , T) = \epsilon _{\infty }-\frac{c^{2}}{\omega ^{2}{\lambda _{L}}^{2}(T)}- \frac{{{\omega _{p}}^{2}}{\tau ^{2}}}{1+{\omega ^{2}}{\tau ^{2}}}f_{n}+i \frac{{\omega _{p}}^{2}\tau }{\omega (1+{\omega ^{2}}{\tau ^{2}})}f_{n}. \end{aligned}$$

Finally, substitution of Eq. (8) leads to the dielectric function of a metal

10 $$\begin{aligned} \epsilon (\omega ) = \epsilon _{\infty }-\frac{{\omega _{p}}^{2}}{\gamma ^{2}+\omega ^{2}}+i \frac{{\omega _{p}}^{2}\gamma }{\omega (\gamma ^{2}+\omega ^{2})}. \end{aligned}$$

To calculate the reflectance spectra, in our numerical simulations layer 1 corresponds to ${\text {Ba}}{\text {Ti}}{\text {O}}_{3}$ with $a=30\, {\hbox {nm}}$; $\epsilon =5.8$^77^. As the operation temperature in the present experiment varies from 20 K to 100 K, the ${\text {Ba}}{\text {Ti}}{\text {O}}_{3}$-dielectric constant can be considered constant in this range^78,79^. Layer 2 corresponds to ${\text {Y}}{\text {Ba}}_{2}{\text {Cu}}_{3}{\text {O}}_{7}$, with $p=2$, $T_{c}=80\, {\hbox {K}}$ (the critical temperature experimentally obtained in this work), $b=73\, {\hbox {nm}}$, the plasma frequency $\omega _{p}=1.7 \times 10^{15}\, {\hbox {rad/s}}$^80^, the damping frequency $\gamma =1.3 \times 10^{13}\, {\hbox {rad/s}}$^80^, and the dielectric constant is modelled by Eq. (9). According to the growth direction of the bilayers (c-axis), we consider the parallel propagation to the c-axis of ${\text {Y}}{\text {B}}{\text {C}}{\text {O}}$, i.e., the magnetic field $\mathbf {H}$ perpendicular to ${\hat{c}}$ (TE case). Therefore, the penetration depth is on the a–b plane with a $\lambda _{\perp 0}=118.6\, {\hbox {nm}}$^81^. For temperatures close to the critical temperature $T_{c}$, over the range $0.001< \frac{T_{c}-T}{T}< 0.1$, we adopted the magnetic penetration depth ${ \lambda }_{ L }=\lambda _{\perp 0}[1-(T/T_{c})]^{p}$, with $p=-\frac{1}{3}$^81^. Simultaneously, we considered the thermal expansion effect on ${\text {Ba}}{\text {Ti}}{\text {O}}_{3}$ and ${\text {Y}}{\text {Ba}}_{2}{\text {Cu}}_{3}{\text {O}}_{7}$ thicknesses. In certain temperature ranges, the thermal expansion effect adopts the law $d(T)=d_{0}(1+\alpha \Delta T)$, where $\alpha$ is the thermal expansion coefficient, $\Delta {T}$ is the temperature deviation, and *d* and $d_{0}$ are the thicknesses of each layer under actual and room temperature, respectively^82^. We consider the thermal expansion coefficients to be $7.14 \times 10^{-6} /^{\circ } {\hbox {C}}$ and $13.4 \times 10^{-6} /^{\circ } {\hbox {C}}$ for ${\text {Ba}}{\text {Ti}}{\text {O}}_{3}$^83^ and ${\text {Y}}{\text {Ba}}_{2}{\text {Cu}}_{3}{\text {O}}_{7}$^84^ , respectively.

The steady-state *dc* conductivity ($\sigma _{0}$) and *dc* resistivity ($\rho _{0}$) are functions of the plasma frequency $\omega _{p}$ and the damping $\gamma$, and they are related as follows^72^

11 $$\begin{aligned} \sigma _{0} = \frac{1}{\rho _{0}}=\frac{nq^{2}\tau }{m}=\frac{\epsilon _{0}{\omega _{p}}^{2}}{\gamma }. \end{aligned}$$
